# A global database of bird nest traits

**DOI:** 10.1038/s41597-023-02837-1

**Published:** 2023-12-21

**Authors:** Stephanie Yuan Chia, Yi-Ting Fang, Yi-Ting Su, Pei-Yu Tsai, Chia Hsieh, Shu-Han Tsao, Jia-Yang Juang, Chih-Ming Hung, Mao-Ning Tuanmu

**Affiliations:** 1 Biodiversity Research Center, Academia Sinica, Taipei, Taiwan; 2 Department of Biology, University of Maryland, College Park, Maryland USA; 3 Department of Life Sciences, National Cheng Kung University, Tainan, Taiwan; 4 Program in Ecology and Evolutionary Biology, BioSciences Department, Rice University, Houston, Texas USA; 5 Department of Mechanical Engineering, National Taiwan University, Taipei, Taiwan; 6 Program in Nanoengineering and Nanoscience, Graduate School of Advanced Technology, National Taiwan University, Taipei, Taiwan

**Keywords:** Macroecology, Evolutionary ecology, Behavioural ecology

## Abstract

The reproductive success of birds is closely tied to the characteristics of their nests. It is crucial to understand the distribution of nest traits across phylogenetic and geographic dimensions to gain insight into bird evolution and adaptation. Despite the extensive historical documentation on breeding behavior, a structured dataset describing bird nest characteristics has been lacking. To address this gap, we have compiled a comprehensive dataset that characterizes three ecologically and evolutionarily significant nest traits—site, structure, and attachment—for 9,248 bird species, representing all 36 orders and 241 out of the 244 families. By defining seven sites, seven structures, and four attachment types, we have systematically classified the nests of each species using information from text descriptions, photos, and videos sourced from online databases and literature. This nest traits dataset serves as a valuable addition to the existing body of morphological and ecological trait data for bird species, providing a useful resource for a wide range of avian macroecological and macroevolutionary research.

## Background & Summary

The architecture of a nest weaves the story of avian survival and adaptation. The remarkably diverse nest designs, from simple scrapes on the ground, piles of sticks floating on the water, to intricately woven chambers hanging in trees, reflects various strategies employed by birds to optimize their reproductive success in diverse biotic and abiotic environments^1,2^. Certain nest building behaviors, such as placing nests high up in trees or cliff crevices, constructing dome or cavity nests, and suspending nests from drooping twigs, are believed to mitigate the risk of nest predation^3–5^. Enclosed nests are also thought to provide better thermoregulation properties, offering protection against extreme temperatures^6–8^. Conversely, the versatility and efficiency of building open-cup nests are believed to facilitate the expansion of ecological niche^9^. Furthermore, nest site selection and the complexity of nest forms can reflect the learning abilities and manipulation skills of bird species^10,11^. These findings underscore the potential significance of nest traits in improving our understanding of how birds evolve and adapt to diverse environments.

There is indeed a growing interest in using nest traits to explore the evolutionary mechanisms of other life-history traits and behaviors of bird species^5,12–14^. Nest traits are also being used to investigate the environmental influences on avian species distribution and community compositions^15–17^. However, these studies have been limited in their spatial or species coverage due to the absence of comprehensive and structured nest traits information. In addition, inconsistent definitions of nest traits across studies have made it difficult, if not impossible, to integrate information from different studies. To fill this gap, we conducted a thorough characterization of nest structure, nesting site, and attachment approaches for over 9,200 bird species worldwide, compiling them into a structured and readily accessible dataset.

The dataset is distinguished from currently available data^9,12,16,17^ in its uniqueness, uniformity, completeness, and cross-referencing capability. Firstly, instead of employing simplistic categorizations such as open/closed or ground/off-ground nests^3,18^, our dataset provides detailed information on multiple dimensions of nest characteristics, which exhibit distinct but interdependent evolutionary trajectories throughout the avian phylogeny^19^. Thus, including multiple nest traits in research can provide a more holistic context for understanding the avian behaviors. Secondly, by implementing a rigorous procedure and extracting information from reputable data sources, we strived to ensure the quality and consistency of the dataset. Thirdly, we managed to characterize nest traits for the vast majority of bird species, making it applicable to studies in a broad range of geographical and taxonomic scopes. Lastly, we provide multiple species identifiers that enable easy integration with existing information, such as species’ geographic distribution^20^, phylogeny^21^, and morphological, ecological and life history traits^22,23^.

## Methods

### Definition of nest characteristics

We conducted a characterization of bird nests, focusing on three ecologically relevant features: site, structure and attachment, as outlined by Fang *et al*.^19^ The nest site refers to the specific location where nests were constructed, and we classified them into seven distinct types: ground, tree, non-tree vegetation, cliff or bank, underground, termite or ant nest, and waterbody (Table 1). In our dataset, the term “tree” indicates a woody plant with an elongated stem and secondary growth. Other tall plants that lack secondary growth, including monocots like palms, banana trees, and bamboos, as well as tree ferns, are classified as non-tree vegetation. Nests constructed within climbing vines or epiphytes growing on trees are also categorized as non-tree vegetation. We considered a site as a waterbody only when the nest was floating on the water surface or built up from the bottom of the water. Nests located in plants emerging from the water (e.g. reeds, mangroves) or on tree branches above the water surface were not included in this particular category.

**Table 1:** Description of nest site types.

Type	Definition
Ground	Nests physically in contact with the ground or only a few centimeters above the ground on a cushion of vegetation
Tree	Nests built in/on any part of a tree (a woody plant with an elongated stem and secondary growth), such as branches, stem, and stump, including dead and fallen trees
Non-tree vegetation	Nests built in non-tree vegetation such as bushes, thick tangled herbaceous vegetation (such as vines or reeds), ferns, bamboo and palms
Cliff or bank	Nests built in/on cliffs, river banks, or piles of soil or rocks
Underground	Nests built in burrows underground
Termite or ant nest	Nests built in termite or ant nests
Waterbody	Nests floating on the surface of water or piled up from the bottom of water bodies and thus surrounded by water

**Fig. 1:**
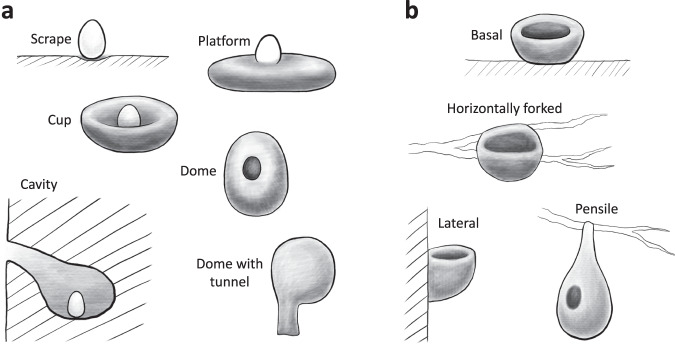
Illustrations of (**a**) seven nest structures and (**b**) four nest attachment types defined in our dataset. It is important to note that while the illustrations provide a typical example for each nest type, variations exist within each category that are not explicitly depicted here.

To describe the nest structures, we followed the modified definitions from a previous study^24^ and identified seven types: scrape, platform, cup, simple dome, dome with tunnel, primary cavity, and secondary cavity (Table 2 and Fig. 1a). Nests built inside cavities were classified as cavity nests, regardless of any additional structures (such as platforms, cups, or domes) constructed within the cavity.

**Table 2:** Description of nest structure types.

Type	Definition
Scrape	Nests with no obvious construction, with only brief scratching or cleaning, or with only basic linings such as leaves or stones, often described as scrapes, or depressions on ground or cliffs
Platform	Nests constructed by stacking or loosely intertwining materials such as feathers, leaves, sticks, or vines to form a platform or pad
Cup	Nests with an elevated rim made of interweaving nest materials or mud but cannot fully conceal the body of parental birds, commonly described as cups, or sometimes saucer. This category also includes half-cup shaped nests attached to walls.
Simple dome	Nests woven into an enclosed structure that allows parental birds to sit inside without any part of their bodies exposed. Such a nest may be described as a ball-, spherical-, or oval-shaped nest, dome, semi-dome, purse, or cavity built with soft materials such as grasses.
Dome with tunnel	Dome nests with a tunnel- or tube-entrance. A dome nest with projected structure(s) that are not used as an entrance (e.g. tail) is categorized as a simple dome rather than a dome with tunnel.
Primary cavity	Nests in a cavity excavated by the nesting birds themselves
Secondary cavity	Nests in a cavity that was formed naturally or initially excavated by other animals, with or without modifications to the existing structure. Such cavities can be described as holes, crevices in walls or rocks, or burrows.

Nest attachment refers to how nests are connected to supporting objects. We categorized the attachment types as basal, horizontally forked, lateral, and pensile attachment (Table 3 and Fig. 1b). Notably, we distinguished nests laterally attached to vertical objects (e.g. walls or stems) as a separate category from those supported by horizontally forked branches, as they likely involve different building techniques.

**Table 3:** Description of nest attachment types.

Type	Definition
Basal	Nests mainly supported from the bottom, including those sitting among multiple interweaving tree or bush branches and those inside cavities
Lateral	Nests attached to one or more vertical supporting objects by their lateral parts, such as those attaching to walls, or built between vertical stems of tall grasses.
Horizontally forked	Nests attached to two or more horizontal branches by their lateral parts
Pensile	Nests hanged down from a supporting object with their upper and narrower parts attached, often described as pendant, suspending, or hanging from the supporting structure.

Certain nesting situations were not adequately represented by the predefined nest types. For species within the Megapodiidae family that construct mounds and use the solar or fermentation heat for egg incubation, we established a distinct category called “mound”. Obligate brood parasitic species which do not build their own nests but rely on other species to raise their young were classified separately as “parasite”. Note that the nest characterization in our dataset only covers natural environments and structures. We have not included artificial structures used for nest building due to the limited availability of comprehensive documentation in this regard.

### Data sources

We gathered information on nest characteristics from various forms of sources, including text descriptions, photos, and videos. The primary source for a significant portion of the dataset was the Handbook of the Birds of the World (HBW) Alive, which is now integrated into the online platform Birds of the World^25^. This comprehensive database provided extensive life history information for most bird species. We extracted nest-related information from the text descriptions in the breeding section of the species, including details on nest site characteristics, nest construction process, nest structure and size, nesting materials, supporting materials and the nest attachment mechanisms.

To supplement our characterization efforts, we also used alternative data sources, including (1) photos, videos, and references embedded or cited in the description of the bird species in HBW Alive (Birds of the World), (2) large online database managed by reputable organizations engaging in long-term bird data collection, including the Macaulay Library from The Cornell Lab of Ornithology (www.macaulaylibrary.org), National Audubon Society (www.audubon.org), WikiAves (www.wikiaves.com.br), and BirdLife Data Zone (https://www.datazone.birdlife.org), (3) other online data sources, including websites engaging in smaller-scale data collections, and informal sources such as personal blogposts, photos, and videos, and (4) published scientific literature on breeding behavior of the species, retrieved from Google Scholar using the scientific name of the species with keywords “nest” or “breeding”.

When the text descriptions of bird nests in HBW Alive (Birds of the World) were missing or incomplete, we turned to the alternative references mentioned above, following a prioritized order outlined in Table 4. If nest information remained unavailable, we referred to species with similar nesting behavior, if indicated in the HBW Alive (Birds of the World) descriptions, and recorded the scientific name of the reference species. In the case of recently split species without their own information page in HBW Alive (Birds of the World), we referred to the species prior to the split.

**Table 4:** Code for different types of references.

Code	Description	Priority
H	HBW Alive (Birds of the World)	1
E	Embedded photos and videos and references cited in HBW Alive (Birds of the World)	2
D	Large online databases managed by reputable organizations	3
O	Small online databases or informal online data sources such as personal blogposts, photos, and videos	4
L	Scientific literature	5
S	Refer to the nest characters of similar species as indicated in HBW Alive (Birds of the World)	6
N	Not available	7

### Data extraction procedure

We obtained the list of the world’s bird species from HBW and BirdLife Taxonomic Checklist Version 5.0^26^, selecting taxonomic entities that were recognized as species (BirdLife taxonomic treatment = R), resulting in a total of 11,158 candidate species for nest characterization. To enhance data integration across bird trait datasets, we included two species identifiers, SISRecID and Avibase ID, alongside the scientific name of each species. We also included the order, family, and common name for each species, facilitating convenient data processing tasks such as grouping and subsetting.

To code nest characteristics for each species, we created a spreadsheet with species as entities (rows) and the defined nest types (7 types of site, 7 types of structure, 4 types of attachment) as attributes (columns). Each data entry was assigned a value of 1 if the corresponding nest characteristic was observed in the species, or 0 if not observed. This approach allowed us to capture multiple nest types for species that exhibited more than one type of site, structure, or attachment, as described in the references. Mound-building species were assigned a value of 1 to the *mound* column. Brood parasitic species that do not build nests were assigned 1 to the *parasite* column and 0s to all other nest type columns. Several species in the Falconidae family that do not build nests but only use pre-existing nests from other bird species do not fall within any of the categories and were therefore assigned 0s to all nest type columns. When information was missing or insufficient for identifying the nest types, NAs were assigned to the respective columns.

Due to the extensive amount of information to be collected and verified, the characterization task spanned from October 2016 to September 2023 and involved six researchers. For each species, we documented the data sources associated with each nest trait, the name(s) of the researcher(s) responsible for the identification, and the reference period during which the data was collected. For data sourced from large online databases (coded D in Table 4), we recorded the main website from which the species information can be searched with its scientific name. For data sourced from other online data sources (code O) or scientific literature (code L), we recorded the specific webpage or literature directly associated with the respective species. For data sourced from HBW Alive (Birds of the World) page of similar species (code S), we provided the scientific name of the corresponding similar species.

## Data Records

The nest trait dataset is available with three comma-separated values (CSV) files on Zenodo^27^. The main file with the name NestTraits_v2.csv documents the nest traits of the world’s bird species. The supportive file named NestTraits_v2_metadata.csv includes the descriptions of columns and data entry codes used in the main file. The other supportive file named NestTraits_v2_ref.csv includes a list of references used to characterize nest traits in our dataset. We also present here the column descriptions in Table 5.

**Table 5:** Column descriptions.

Colum name	Description	Data type	Values
Seq_HBWBLv5	Sequence number of the HBW-BirdLife version 5.0 species checklist	Integer	—
Scientific_name	Scientific name of the species	Character	—
Order	Order of the species	Character	—
Family	Family of the species	Character	—
Common_name	Common name of the species	Character	—
SISRecID	Taxonomic identifier used by IUCN/BirdLife International	Character	—
AvibaseID	Taxonomic identifier assigned by Avibase	Character	—
Parasite	Whether being an obligate brood parasite	Character	0 = no; 1 = yes; NA = Not available
Mound	Whether building a mound nest	Character	0 = no; 1 = yes; NA = Not available
NestSite_ground	Whether building a nest on the ground	Character	0 = no; 1 = yes; NA = Not available
NestSite_tree	Whether building a nest in/on trees	Character	0 = no; 1 = yes; NA = Not available
NestSite_nontree	Whether building a nest in/on non-tree vegetation	Character	0 = no; 1 = yes; NA = Not available
NestSite_cliff_bank	Whether building a nest on cliffs or banks	Character	0 = no; 1 = yes; NA = Not available
NestSite_underground	Whether building a nest underground	Character	0 = no; 1 = yes; NA = Not available
NestSite_waterbody	Whether building a nest in/on water bodies	Character	0 = no; 1 = yes; NA = Not available
NestSite_termite_ant	Whether building a nest in termite or ant nests	Character	0 = no; 1 = yes; NA = Not available
NestStr_scrape	Whether building a scrape nest	Character	0 = no; 1 = yes; NA = Not available
NestStr_platform	Whether building a platform nest	Character	0 = no; 1 = yes; NA = Not available
NestStr_cup	Whether building a cup nest	Character	0 = no; 1 = yes; NA = Not available
NestStr_dome	Whether building a dome nest	Character	0 = no; 1 = yes; NA = Not available
NestStr_dome_tunnel	Whether building a dome nest with a tunnel	Character	0 = no; 1 = yes; NA = Not available
NestStr_primary_cavity	Whether building a primary cavity nest	Character	0 = no; 1 = yes; NA = Not available
NestStr_second_cavity	Whether building a secondary cavity nest	Character	0 = no; 1 = yes; NA = Not available
NestAtt_basal	Whether building a nest with basal attachment	Character	0 = no; 1 = yes; NA = Not available
NestAtt_forked	Whether building a nest with horizontally forked attachment	Character	0 = no; 1 = yes; NA = Not available
NestAtt_lateral	Whether building a nest with lateral attachment	Character	0 = no; 1 = yes; NA = Not available
NestAtt_pensile	Whether building a nest with pensile attachment	Character	0 = no; 1 = yes; NA = Not available
Ref_site	Reference code for nest site characterization	Character	codes described in Table 4
Ref_str	Reference code for nest structure characterization	Character	codes described in Table 4
Ref_att	Reference code for nest attachment characterization	Character	codes described in Table 4
Identifier	The last name of the identifier(s)	Character	—
Retrieval_time	Time period of nest information retrieval	Character	[yyyy MMM~yyyy MMM]

**Fig. 2:**
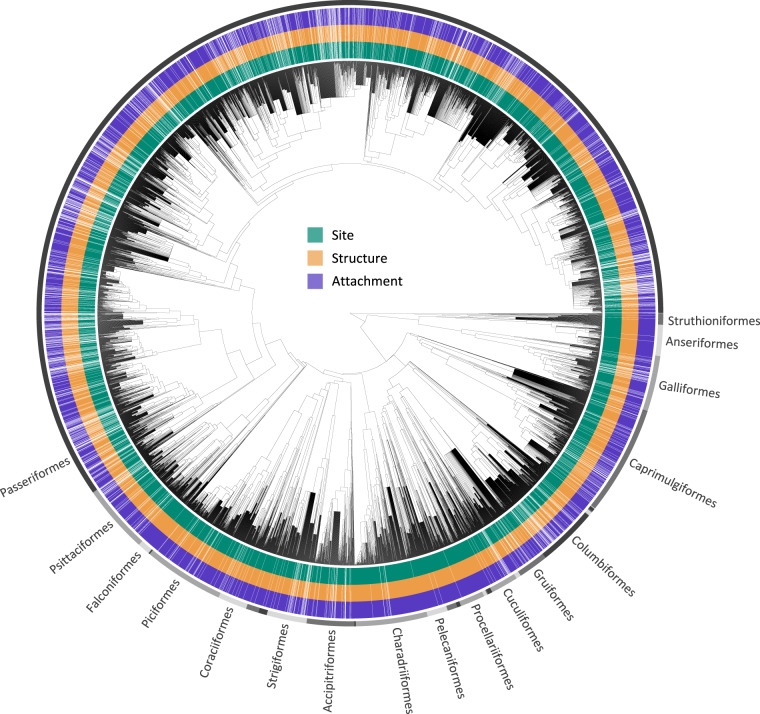
Phylogenetic distribution of nest characteristics data at the species level. The three inner colored circles represent the presence of species-level data for each of the three nest characteristics, where white strips indicate missing data. The outer gray colored circle represents the positions of the bird orders on the phylogenetic tree. The figure shows 9,986 species from our dataset that can be matched to the BirdTree taxonomy. The phylogenetic tree was generated using 1,000 trees from BirdTree.org^21^ with the majority rule for tree topology and least squares method for edge lengths using R package *phytools*^30^. The diagram was generated using R package *ggtree*^31^.

Excluding the missing data, the presented dataset covers bird species from all 36 orders, more than 98% of the 244 families, more than 92% of the 2,379 genera, and over 81% of the 11,158 species (Table 6). These species are well-distributed across the avian phylogenetic tree (Fig. 2), and the dataset covers more than 71% of the species in each of the 36 orders, ensuring substantial coverage within various lineages.

**Table 6:** Data coverage at different taxonomic ranks.

Taxonomic rank	Site	Structure	Attachment	Total
Species	9,113 (81.7%)	9,026 (80.9%)	8,976 (80.4%)	11,158
Genus	2,228 (93.7%)	2,214 (93.1%)	2,214 (93.1%)	2,379
Family	241 (98.8%)	241 (98.8%)	241 (98.8%)	244
Order	36 (100%)	36 (100%)	36 (100%)	36

**Fig. 3:**
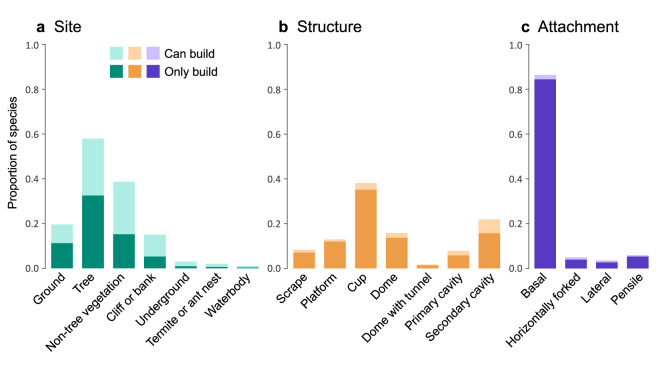
Frequency distribution of nest types across avian species. The bars indicate the proportion of species using the specific type of nest site, nest structure, or nest attachment. A dark-colored bar refers to species building nests of only the focal type, whereas a lighter-colored bar refers to additional species building multiple types of nests including the focal type. Species without available data were excluded from the frequency calculation.

The majority of species utilize vegetation as nesting sites. More than half (58%) of the species can nest in/on trees, and more than one third (39%) of the species can nest in/on non-tree vegetation. Apart from that, there is a considerable number of ground-nesting species (19%) and species that can nest in cliffs or banks (15%). The other types of sites, i.e., underground, termite or ant nests, and waterbodies, were used by a relatively small proportion of species (<3%). As for the nest structure, the most common type is the cup-shaped nest, which can be built by 38% of the species. Meanwhile, other nest structures, including dome, platform, scrape, and primary and secondary cavities, were all found in more than 7% of the species, revealing the diverse nest structures among species. Regarding nest attachment, basal attachment is the most common type and used by 86% of the bird species, with the other three types ranging only from 3% to 6%. The frequency distribution of each nest type can be found in Fig. 3.

## Technical Validation

To minimize inconsistencies and potential errors in the characterizing process, we implemented several validation procedures. Firstly, when encountering ambiguous text description or atypical nest forms, two or more researchers reviewed and discussed the cases until a consensus was reached. This ensured that judgments were made collectively, reducing the likelihood of inconsistency. Secondly, we conducted random checks to validate the final results. We selected random species from each nest type category to check for correctness and consistency. If systematic errors were found associated with specific nest types, taxa, or researchers, we would review other relevant species to trace and correct the errors. Thirdly, we compiled the nest characteristics data into a site-structure-attachment combination occurrence table, focusing on rare or unusual combinations of nest type. We then revisit the data sources to verify the entries of these species. Fourthly, we summarized the distribution of nest types for each of the 35 non-passerine orders and 139 passerine families, and for species having nest types that were uncommon in their order or family, we verified the data entries for the species by revisiting the data sources. For families containing only one or very few species, all species were double-checked at least once. Among the total species examined, a minimum of 3,930 (35.2%) species were verified by two or more researchers across all trait entries, not including a larger number of species that were partially verified for specific nest traits.

We implemented measures to facilitate corrections and future maintenance of the dataset. For each species, we recorded the type of references used for trait identification for each species, which enabled verification in case of doubts. We also recorded the time period of data extraction, which can be used to prioritize the updating efforts when new information is available. Additionally, we recorded the names of the researchers responsible for identifying the traits of each species. This allows for efficient identification and correction of systematic errors made by specific individuals.

## Usage Notes

### Taxonomic identifiers

**Fig. 4:**
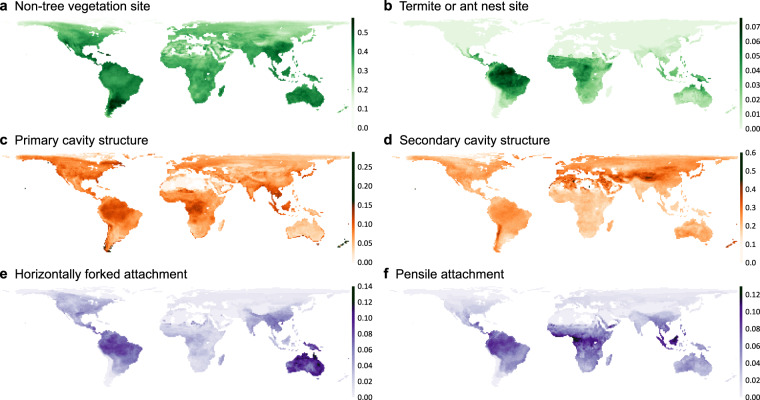
Global distributions of specific nest site, structure, and attachment types. Two nest types for each of the three nest traits are illustrated: nest sites in (**a**) non-tree vegetation and (**b**) termite or ant nests, nest structures of (**c**) primary cavities and (**d**) secondary cavities, and nest attachments of (**e**) horizontally forked and (**f**) pensile forms. The color darkness indicates the proportion of species within the local assemblage that build the respective nest type. The species distribution data used, representing resident and breeding ranges, were obtained from BirdLife International and HBW^20^. The figures have a spatial resolution of ~1 degree with an equal area projection. Values of grid cells with less than ten species were excluded and shown in white. The maps were generated using R packages *raster*^32^ and *rgdal*^33^.

The nest trait dataset provided several commonly used species identifiers to facilitate seamless referencing between different datasets, eliminating the complexities associated with cross-referencing across various species naming systems. The HBW-BirdLife version 5.0 checklist’s sequence number can be used to link to other species information within the original checklist, such as IUCN Red List category, synonyms, and subspecies. The SISRecID provides a means to connect with previous versions of HBW and HBW-BirdLife species checklists. This would be useful to link to datasets with earlier versions of nomenclature, which is used in many published datasets such as EltonTraits^23^ and BirdLife International species distribution data^20^. For example, a linkage between the current dataset and species range maps from BirdLife International can provide intriguing geographical patterns presenting various opportunities for further research in avian ecology, biogeography, and species interactions (Fig. 4). Additionally, the Avibase ID^28^ enables linking to other taxonomies such as Clement/eBird taxonomy^29^, and allows for tracking changes in the scientific names.

### Filling in missing data

As nest characteristics often exhibit strong phylogenetic signals and are generally conserved within a genus, users have the option to supplement missing data by referring to information from species within the same genus. This approach can potentially increase species coverage from 80–82% in the current dataset to 98%. In situations where variations in nest types exist within a genus, it is up to the users to determine the protocol for filling in the traits of particular species. This may involve choosing the most commonly observed type, the strictly shared type, considering all possible types, or referencing data from the closest related species (if available), depending on the specific goals of the analysis. Similar procedures can be extended to higher taxonomic levels, such as the family or even order, to further increase species coverage.

### Limitations

We acknowledge that the identification of nest traits is subject to several limitations and constraints. The reliance on text descriptions for identification introduces a degree of sensitivity to the perception and wording of the original describer of the nests. Ambiguity in the description can also add a level of subjectivity in the interpretation by our nest characterization team. In addition, for species with limited observation records, the nest description may be based on a single or very few observations, potentially limiting the comprehensive representation of nest traits. Conversely, species that have been extensively documented may exhibit a wider range of nest forms in the dataset. Due to these challenges, we recognize the potential for future updates to enhance the completeness and accuracy of the dataset as new information becomes available.

## Data Availability

No custom code was used.
